# Clipping of Posterior Circulation Intracranial Aneurysms: Maintenance of a Necessary Skill in Low-Resource Settings

**DOI:** 10.7759/cureus.19170

**Published:** 2021-10-31

**Authors:** Panduranga Seetahal-Maraj, Narindra Ramnarine

**Affiliations:** 1 Neurosurgery, San Fernando General Hospital, San Fernando, TTO; 2 Clinical Surgical Sciences, The University of the West Indies, St. Augustine, TTO

**Keywords:** endovascular therapy, posterior circulation aneurysm, low-resource setting, surgical clipping, posterior inferior cerebellar artery, intracranial aneurysm

## Abstract

The treatment paradigm for intracranial aneurysms has evolved with technological advancements, resulting in improved patient outcomes. In particular, the management of posterior circulation aneurysms has shifted to favor endovascular therapy (EVT). However, this modality is not always accessible in low-resource settings.

In our country (Trinidad and Tobago), neuroendovascular services are not readily available. We report a case of a patient with a ruptured left posterior inferior cerebellar artery (PICA) aneurysm (Fisher grade 4) who underwent a far-lateral craniotomy and clip ligation. It was done by a fellowship-trained vascular neurosurgeon in a public hospital and resulted in an excellent patient outcome. This highlights the need to maintain this surgical skill set in resource-poor countries, in spite of the increasing popularity of endovascular therapy.

## Introduction

Treatment paradigms for intracranial aneurysms have changed with technological advancements, resulting in improved patient outcomes. The management of posterior circulation aneurysms has shifted toward favoring endovascular therapy (EVT) [1]. However, this modality is not always accessible in low-resource settings. On our island (Trinidad and Tobago), neuroendovascular services are not available in the primary healthcare systems and are extremely costly in a select few private institutions. This, unfortunately, excludes most of the population from accessing it, and classic microsurgical strategies need to be implemented in the treatment of intracranial aneurysms.

We report a case of a patient with a ruptured posterior inferior cerebellar artery (PICA) aneurysm (Fisher grade 4) who underwent a far-lateral craniotomy and clip ligation with a good outcome. This highlights the need to maintain the ability to surgically clip aneurysms, especially in countries where endovascular therapy is not readily available.

## Case presentation

A 55-year-old male presented to the emergency department with a thunderclap headache after sexual intercourse. He denied any nausea/vomiting, fever, seizures, or visual disturbance. His past medical history revealed no comorbidities. He was hemodynamically stable, with a pulse of 92 beats per minute and blood pressure of 158/85 mmHg. On neurological examination, his Glasgow Coma Scale (GCS) score was 15/15, and both pupils were 3 mm and reactive to light and accommodation. His sensorimotor examination was normal, but a positive Brudzinski's sign was elicited, indicating meningismus. Both of his plantar reflexes were downgoing. A computed tomography (CT) scan of the brain was done, which revealed diffuse subarachnoid hemorrhage, with blood in the third and fourth ventricles and a heavy blood load in the perimesencephalic cisterns. The subarachnoid hemorrhage was graded as Hunt and Hess 1 and Fisher CT grade 4.

**Figure 1:**
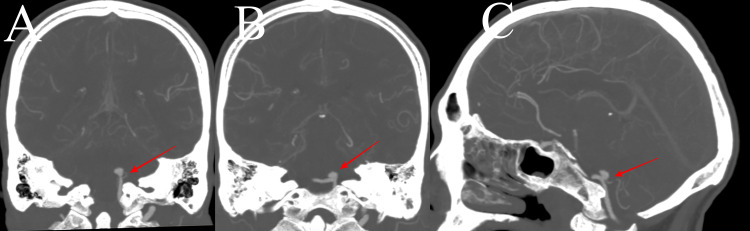
Computed tomography angiogram of the brain Coronal sections showing the origin of the aneurysm from the left posterior inferior cerebellar artery (PICA) takeoff (A, red arrow) and the location in relation to the occipital condyle (B, red arrow). (C) shows a sagittal section of the aneurysm in relation to the left vertebral artery and PICA (red arrow). The course of PICA can be appreciated.

The patient was diagnosed with a likely ruptured posterior circulation aneurysm and subsequently referred to the neurovascular service at our hospital. No catheter laboratory was available at our institution (a public hospital), and the cost was prohibitive at private facilities. We opted for CT angiography, which confirmed a 5 x 4 x 5 mm left posterior inferior cerebellar artery (PICA) aneurysm (Figure 1). The left vertebral artery was dominant, and the right was severely hypoplastic. The patient did not develop hydrocephalus and remained hemodynamically stable. The management strategy included maintaining systolic blood pressures in a range of 120-160 mmHg and commencing nimodipine in order to reduce the risk of vasospasm.

Since endovascular therapy was not a possible option for this patient, he was counseled on surgical clipping via a far-lateral craniotomy. Aside from a high-speed drill, operative microscope and a limited selection of aneurysm clips (Sugita, cobalt alloy), no adjuncts commonly used in aneurysm surgery were available. This included neuromonitoring, micro-Doppler, or indocyanine green. Ideally, this patient should have been admitted to a high-dependency unit (HDU) at a minimum, but there were no available beds. It would also be necessary to monitor the patient postoperatively in an HDU setting, and this therefore delayed surgical clipping until day seven post-admission, when one became available.

**Figure 2:**
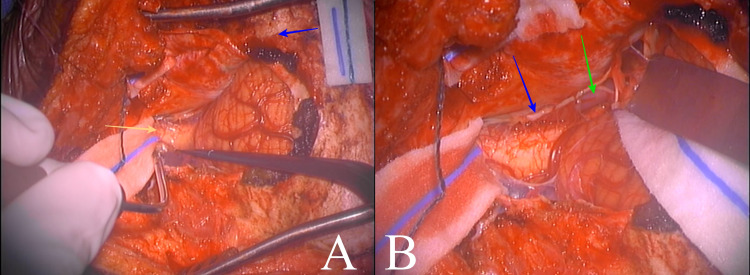
Intraoperative exposure (A) shows far-lateral craniotomy with extension to transverse-sigmoid sinus junction (blue arrow) and cisterna magna (yellow arrow). (B) shows left spinal accessory nerve (blue arrow) and left vertebral artery (green arrow).

**Figure 3:**
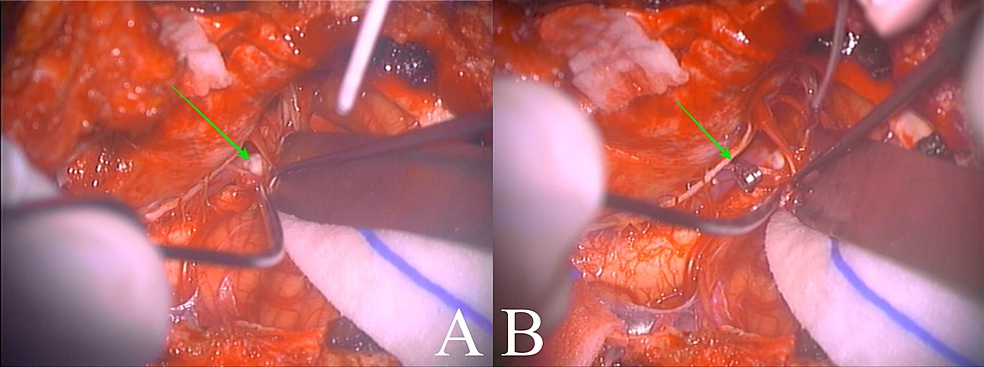
Intraoperative PICA aneurysm exposure (A) shows aneurysm sac (green arrow), and (B) shows the placement of a temporary clip on the proximal vertebral artery (green arrow).

**Figure 4:**
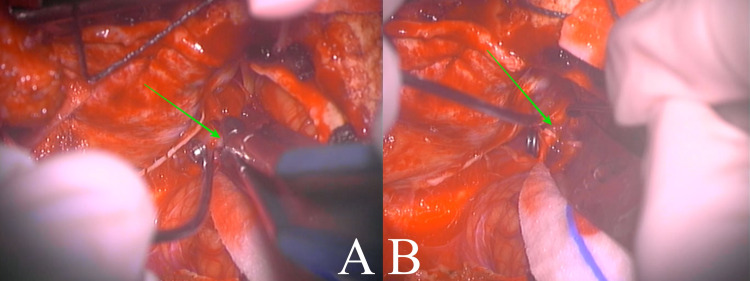
Intraoperative clipping of posterior inferior cerebellar artery aneurysm Placement of permanent clip on the neck of aneurysm (A, green arrow) and visual assessment of the patency of the posterior inferior cerebellar artery (B, green arrow).

The patient was taken to the operating room and placed in a park-bench position. A linear incision was made from C3 to the inion, and then, a hockey stick curve was done toward the left mastoid process. A far-lateral craniotomy was performed, with exposure to the transverse-sigmoid sinus junction, followed by a C1 left hemilaminectomy. A neurosurgical microscope was used to identify and expose the left vertebral artery. Proximal control was gained with a temporary clip, and the aneurysm was identified just at the takeoff of PICA (p1 segment), below the vagus nerve (Figure 2). A 4-mm straight clip was placed over the neck, and the aneurysm sac was then confirmed to be deflated (Figure 3). The PICA remained visibly patent (Figure 4); however, there was no indocyanine green nor micro-Doppler available to confirm otherwise. The procedure was completed, and the patient awoke with no neurological deficits. He was discharged on postoperative day five, and there were no complications. At two-month follow-up, the patient remains neurologically intact.

## Discussion

After the results of the International Subarachnoid Aneurysm Trial (ISAT), there has been a progressive trend toward endovascular therapy (EVT) of intracranial aneurysms [1,2]. This has been compounded by the increasing incidental discovery of unruptured aneurysms. While there are clear benefits to EVT, the cost can be substantial. One study in 2009 found the cost of EVT (coiling) to be $5080 USD, compared to clipping at $3127 USD [3]. Particularly in low- and middle-income countries, this option may be unrealistic [4]. In our setting (Trinidad), EVT is only available at select private institutions. Thus, the majority of the population seek neurosurgical care in government-funded public hospitals, at which there is no capacity for EVT in intracranial aneurysms.

Early meta-analyses of clipping versus coiling showed that coiling did provide better clinical outcomes than clipping. This was more pronounced in good preoperative grade aneurysms but associated with a higher risk of rebleeding. However, mortality rates at one year did not reveal any significant differences [5]. A meta-analysis on coiling versus clipping of posterior circulation aneurysms in 2019 concluded that neither method was superior to the other in preventing permanent neurologic deficits [6]. One study assessing the clipping of 47 PICA aneurysms had no mortalities and only 2.1% morbidity (one patient) [7].

The surgical management of posterior circulation aneurysms thus remains a necessary skill for neurosurgeons to maintain, especially in a low-resource environment. The proximity to vital cranial nerves and the brainstem can make this seem like a formidable challenge. While these typically may be managed by EVT in high-resource settings, this service is not reliably available to us. Additionally, adjuncts to surgical clipping, including micro-Doppler, indocyanine green, somatosensory and motor evoked potentials, hypothermic circulatory arrest, and papaverine, are also unavailable. This further emphasizes the need for a comprehensive understanding of neurovascular anatomy and the art of microsurgical clipping, and training in this should be a primary focus of any neurosurgical department [8]. As evidenced in our case, certain PICA aneurysms may be safely managed by a far-lateral craniotomy and clipping.

## Conclusions

Despite the growing trend toward endovascular therapy for intracranial posterior circulation aneurysms, this modality is not widely available in resource-poor settings. Neurosurgeons in these areas are therefore required to have both a comprehensive understanding of the microsurgical anatomy of the posterior circulation and be competent at surgical clipping. Our experience with the successful management of a ruptured PICA aneurysm in a low-resource setting highlights the importance of preservation of this skill set.
